# Cerebellar syndromes: clinical observations leading to the recognition of the three types

**DOI:** 10.1055/s-0045-1811727

**Published:** 2025-09-19

**Authors:** Mario Manto, Hiroshi Mitoma

**Affiliations:** 1 Centre Hospitalier Universitaire de Charleroi, Unité des Ataxies Cérébelleuses, Charleroi, Belgium.; 2 Université de Mons, Service des Neurosciences, Mons, Belgium.; 3 Tokyo Medical University, Department of Medical Education, Tokyo, Japan.

**Keywords:** Cerebellum, Cerebellar Ataxia, Vestibular System, Cognitive Dysfunction, Mood dMsorders, Internal Models

## Abstract

Cerebellar syndrome is traditionally categorized into three primary types: cerebellar motor syndrome (CMS), vestibulocerebellar syndrome (VCS), and cerebellar cognitive affective syndrome (CCAS) or Schmahmann syndrome (SS). The first type is subdivided into five elemental features: dysmetria, kinetic tremor, asynergia, adiadochokinesis and dyschronometria. The second is characterized by dysmetria of saccades and jerky pursuit, as well as downbeat nystagmus and gaze-evoked nystagmus. And the third type is associated with a broader spectrum of cognitive and affective symptoms, including impairments in executive function, spatial cognition, language processing and emotional regulation. In its extreme form, cerebellar mutism can also develop during childhood following cerebellar vermis surgery. Recent physiological studies have shed light on the underlying neural mechanisms of these syndromes by identifying a common link of dysfunction within the cerebellum's internal forward model. This is essential to the prediction of the outcomes of motor and cognitive actions and underlines dysmetria as the core common element. Despite the diversity in clinical presentation, cerebellar syndromes can be understood as disruptions of a unified neural mechanism, providing a new framework for better understanding of cerebellar deficits.

## HISTORIC MILESTONES

In his landmark 1969 article published in the *Handbook of Clinical Neurology* , Garcin defined ataxia as “a disturbance of coordination which, quite independently of any motor weakness, alters the direction and extent of voluntary movement and impairs the sustained voluntary or reflex muscle contractions necessary for maintaining posture and equilibrium”. 1

In fact, the term “ataxia” has been used since the mid-nineteenth century and was initially coined mainly to describe syphilis infection. Indeed, in the nineteenth century, tabetic ataxia, resulting from syphilis-related dorsal column degeneration, received greater attention than cerebellar degeneration itself. Based on the observations of patients with that condition, Duchenne introduced the concept of locomotor ataxia and suggested a disruption in coordination between antagonistic muscles. 2

Furthermore, the description of olivopontocerebellar atrophy (OPCA) by Dejerine and André-Thomas in 1900 added momentum to clinical research into cerebellar syndromes. 1 In a series of studies between 1899 and 1913, Babinski provided meticulous description of the symptomatology of cerebellar ataxia, including hypermetria (the most common form of dysmetria) and kinetic tremor. 3 4 These were followed by the comprehensive clinical work of André-Thomas, who provided remarkable description of cerebellar gait disturbances. 5 Holmes was the first to highlight the cerebellocerebellar connections and introduced the concept of disturbances in the speed of movement initiation. 6 By the 1920s, motor syndrome for cerebellar ataxia had been well defined.

Since the 1990s, advancements in neuroanatomy and physiology have significantly expanded our understanding of cerebellar syndromes. The reciprocal and parallel neural connections between the cerebellum and the cerebral cortex, coupled with clinical findings, provided compelling evidence of a link between the cerebellum and cognitive functions 7 ( Figure 1 ). Schmahmann and Sherman 8 further elucidated the clinical importance of this connection by describing a constellation of cognitive impairments associated with cerebellar disorders, which they termed cerebellar cognitive affective syndrome (CCAS) or Schmahmann syndrome (SS). Now, CCAS/SS extends to neuropsychiatric symptoms, in the frontiers of neurology and psychiatry. 9 10

**Source:Figure 1:**
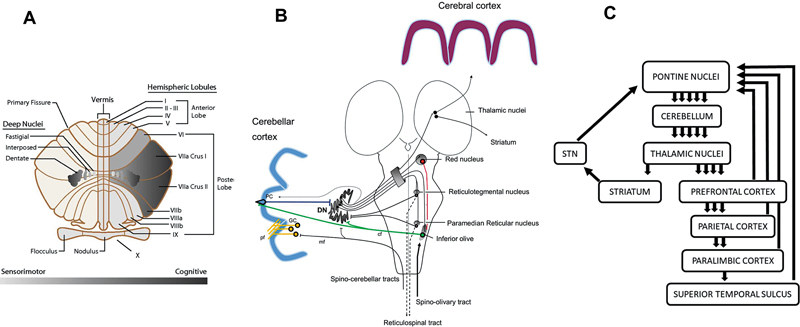
Abbreviations: PC, Purkinje cell; DN, dentate nucleus; cf, climbing fibers; GC, granule cells; pf, parallel fibers; STN, subthalamic nucleus; VTA, ventral tegmental area. Cabaraux et al., 12 with permission. ( **A** ) Cerebellar sensorimotor and cognitive topography on a flattened representation of the 10 lobules. ( **B** ). A corticonuclear projection from the PC to the DN. The DN is involved in dentato-reticular reverberating loops, in the dentato-rubro-olivary triangle (Guillain-Mollaret) and in dentato-thalamo-cortical projections. The cf from the inferior olive project to both the DN and PC via cf. The mossy fibers of the spino-cerebellar tracts target the DN and the GC at the origin of the pf. The afferents to the DN from the locus coeruleus and the raphe nuclei are not illustrated. Both the spinocerebellar tracts and the trigeminocerebellar tracts (not illustrated) relay information from the limbs and head/face, respectively. ( **C** ) Multiple loops running in parallel from the cerebellum to the cerebral cortex and striatum. The striatum projects to the STN, which targets pontine nuclei (disynaptic projection from the STN to the cerebellar cortex). The cerebellum receives not only corticopontine afferents from motor/nonmotor areas, but also information from the superior colliculus, the mammillary bodies and projects toward the VTA, locus coeruleus, and hypothalamus, which are connected with the limbic/paralimbic regions linked to emotional processing (cerebro-cerebellar-limbic loops, not illustrated). The so-called limbic cerebellum engages mostly midline areas of the cerebellum.

Based on this background and recent clinical observations, cerebellar syndromes are now classified into three distinct types: 11 cerebellar motor syndrome (CMS); vestibulocerebellar syndrome (VCS); and CCAS/SS.

Remarkably, neural circuits within the cerebellar cortex exhibit a uniform structure regardless of their location, which is a unique feature in the brain ( Figures 2 3 ). 12 13 The functional units comprising the cerebellar cortex and nuclei are believed to perform identical functions irrespective of the specific area they are coupled to, meaning the particular circuit network they are embedded in, 12 with features of redundancy ( Figures 2 3 ).

**Source:Figure 2:**
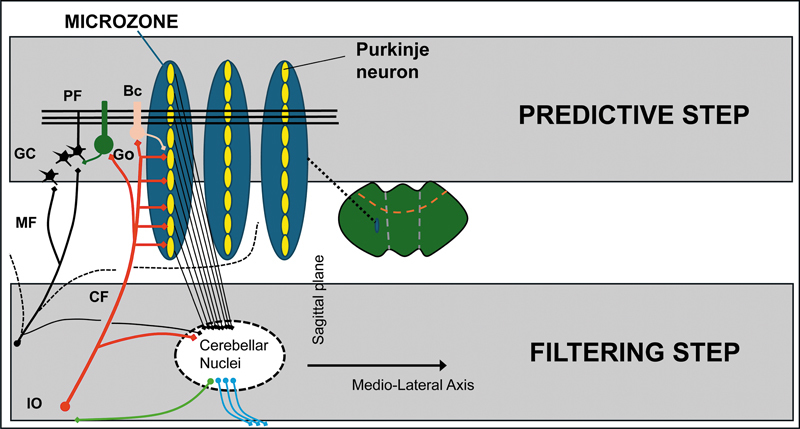
Abbreviations: PF, parallel fiber; CF, climbing fiber; GC, granule cell; Go, Golgi cells; Bc, basket cell; IO, inferior olive. Adapted from Mitoma et al., 13 with permission. A scheme of microzones. A functional congruence between the two major input systems (mossy and climbing fibers) is observed anatomically, with a contribution of mossy fibers into multizonal microcomplexes integrated in cerebellar modules subserving the operational aspects of the cerebellar machinery.

**Figure 3:**
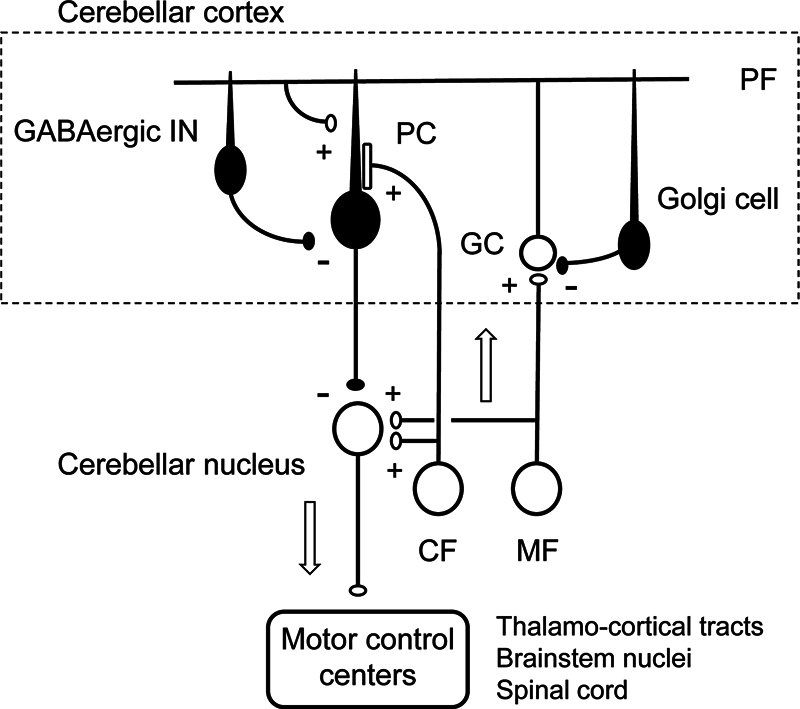
Abbreviations: MF, mossy fiber; CF, climbing fiber; GC, granule cell; PF, parallel fiber; PCs, Purkinje cells; GABAergic IN, stellate and basket cells;cells represented by open circles are excitatory neurons, and their synapses, marked with a +, are excitatory. Cells represented by closed circles are inhibitory neurons, and their synapses, marked with a –, are inhibitory. A scheme of the fundamental cerebellar neural circuit.

In the 1990s, Schmahmann proposed the concept of “dysmetria of thought and emotion”. 14 The hypothesis postulated that, based on the uniform neural circuitry of the cerebello-cortico-cerebellar nuclear complex, a common operational principle governs both the motor and cognitive-emotional domains (universal cerebellar transform). Consequently, similar pathologies arise in disorders that affect either domain. 14 In essence, the cerebellum regulates the speed, consistency, competence, and appropriateness of cognitive-emotional processes, akin to its control over the speed, rhythm, power, and accuracy of motor functions. 15

It is now widely accepted that cerebellar impairments lead to “impaired outcome prediction” not only in the motor domain but also in the cognitive-emotional and vestibular ones. 12 This is a major feature of cerebellar circuitry. Moreover, there is a consensus that the impairment in predictive function stems from a failure of the internal forward model, that is, a failure of the neural mechanism intrinsic to the cerebellum that predicts future control outcomes from current conditions and control signals. 12 The three types of cerebellar syndromes will be described below in light of clinical observations and in the context of internal models.

## CEREBELLAR MOTOR SYNDROME (CMS)

### The five basic elements of CMS

In the *Handbook of Clinical Neurology* , Garcin categorized the various clinical features of cerebellar disorders described by Babinski, André-Thomas, Holmes, and others into five fundamental symptoms. 1 This implies that most cases of cerebellar movement disorders do not manifest a single feature but exhibit diverse characteristics depending on the affected areas and pathological process.

#### *Hypermetria (disturbances in amplitude or metrics of movements)*

This is a well-known phenomenon observed in finger-nose, finger-to-finger/finger-chase, and heel-shin tests. While it is currently termed dysmetria (gathering both hypermetria and hypometria), it was originally known as hypermetria as it represented an overshooting of the target.

Dysmetria is often accompanied by oscillations at the end of movement and is more pronounced at higher speeds or when the inertia of the limb is increased. 16 Defined as an abnormality in the amplitude of movement, it focuses on the failure to reach the target with accuracy. Although a similar phenomenon can be observed in proprioceptive ataxia (interruption of sensory feedback signals due to a peripheral nerve disease or spinal cord lesion), cerebellar ataxia is often characterized by the absence of exacerbation with closed eyes and shows a normal movement direction at the onset of movement.

#### *Kinetic and static tremor (disturbances in continuity of contraction)*

Observed in finger-nose and finger-to-finger tests, this tremor is characterized by discontinuous and intermittent movements rather than smooth and continuous ones. The amplitude of the intermittent ones increases as the patient tries to accurately bring the index finger to the target or as the movement progresses from the starting point to the endpoint. Although termed kinetic tremor, it differs from the regular rest tremor seen in Parkinson's disease due to irregularity and lower frequency. Furthermore, discontinuous and intermittent movements can be observed even during a static state when trying to maintain a posture of the upper limb. Unlike cerebellar dysmetria, kinetic tremor is often reduced by added inertia. Rehabilitation centers usually add mass to the limbs to reduce tremors.

#### *Asynergia (disturbances in combining elementary movements)*

This is defined as an inability to simultaneously integrate multiple movements that constitute a single action. Consequently, each elemental movement appears decomposed. For example, in cerebellar disorders, the upper body may not catch up with the forward movement of the lower limbs during walking. Another example is the failure of lower limb and trunk muscles to cooperate when bending backward simultaneously when performing a backward bending movement in a standing position. This feature is not observed in relatively simple movements, such as finger-nose, heel-shin, and pronation-supination tests, which highlights the need for examination of more complex daily activities.

#### *Adiadochokinesis (disturbances in execution of alternating movements)*

Adiadochokinesis refers to being unable to perform rapidly continuous alternating movements. To test the presence of adiadochokinesis, the patient is asked to pronate and supinate the forearm repeatedly and as fast as possible. In cerebellar disorders, the movements are discontinuous, and each individual movement is prominent.

#### *Dyschronometria (disturbances in speed of initiation and arrest of movements)*

Dyschronometria refers to abnormality in the initiation of movement. For example, when asked to grasp a bar with both hands simultaneously, patients with unilateral cerebellar damage exhibit a delay in the initiation of movement on the affected side. Although not performed routinely in neurological examination, dyschronometria reflects a decrease in facilitation from the cerebellum to the cerebrum and is considered important in classical neurology.

Gait disturbances associated with cerebellar damage are characterized by significant unsteadiness and step irregularity. 17 This feature was described clearly in the 1925 paper of André-Thomas, 5 who stated that the upper body sways significantly forward and backward or side to side. While attempting to maintain balance by widening the gait, the gait becomes zig-zag in nature, with narrow and irregular steps and sudden movements. In this regard, Garcin concluded that these characteristic features are the result of hypermetria and asynergia. 1

In addition to the five fundamental elements defined by Garcin, hypotonia represents another significant symptom of CMS. Muscle tone, in this context, is defined as the resistance encountered during passive stretching of a limb in its relaxed state. 18 Hypotonia can manifest as limb inertia. 1 Patients with cerebellar disorders exhibit a reduced resistance to passive movements and experience excessive amplitudes in such movements. 18

Historically, Luciani characterized the triad of atonia (a reduction in limb resistance to passive manipulation), asthenia (weakness or paresis of movement), and astasia (involuntary oscillations occurring during movement), which occur ipsilaterally to cerebellar lesions in dogs and primates. 19 Holmes later elaborated on the concept of hypotonia, attributing its origin to Luciani's findings. 20 While Holmes studied cerebellar damage arising from gunshot wounds in particular, he noted that hypotonia did not manifest universally. Furthermore, he acknowledged that hypotonia is not a detectable symptom in every type of cerebellar disorder. 18 It tends to be more severe in children with extensive cerebellar lesions.

### Pathophysiologies that unify and explain Garcin's five basic elements: disorders of prediction

Given the diversity of the clinical features of cerebellar ataxia, numerous physiological studies have been conducted to elucidate the underlying pathophysiologies. To analyze the pathophysiology of hypermetria, Hore et al. 21 designed a task that mimicked the finger-nose test, which involved rapid flexion of the elbow joint. He analyzed the associated muscle activity patterns, focusing on agonist-antagonist patterns in the so-called triphasic EMG pattern. In healthy individuals, the biceps muscle is activated first, followed by a decrease in activity and activation of its antagonist, the triceps muscle.

This is because muscle contraction continues slowly even after the cessation of biceps activity and, therefore, the triceps, as an antagonist, contracts to brake the elbow flexion. Importantly, activation of the triceps begins before elbow flexion stretches the triceps. Thus, it is activated in anticipation of the cessation of agonist activity, rather than being a stretch reflex. In patients with cerebellar ataxia, the following abnormalities were observed:

- decreased rate of rise in agonist activity;
- delayed cessation of agonist activity;
- delayed onset of antagonist activity; and
- in the second half of the test, reciprocal alternating activity between the agonist and antagonist muscles.

In other words, dyschronometria and kinetic/static tremor represent delays in the onset of agonist activity. The most important finding is loss of the antagonist's predictive activity (triceps), leading to excessive flexion of the elbow joint due to the overaction of the agonist (biceps), resulting in hypermetria.

Hore's analysis was the first to suggest a link between hypermetria and impaired predictive control. This impairment was confirmed when an inertial mass was affixed to the limb, worsening hypermetria. 16 Cerebellar patients are unable to predict how to scale the intensity of muscle discharges.

The neural mechanisms of the internal model were investigated recently by recording cerebellar Purkinje cell activity in monkeys while they performed tracking tasks. Poppa et al. 22 demonstrated this was both predictive and feedback-based, suggesting the presence of the internal forward model in the cerebellum. Furthermore, Tanaka et al. 23 proposed that this internal forward model is implemented in the cerebellar neural circuit through two stages: predictive and filtering, using the Kalman filter calculations. Thus, there is growing evidence that the fundamental pathology of cerebellar ataxia is a “state of impaired prediction of movement outcome”. 23

The multifaceted characteristics described by Garcin 1 can all be deduced from this principle. To perform coordinated multijoint movements, it is necessary to predict the results from one joint and anticipate the appropriate timing/intensities of activities of several other involved muscles. 12 Therefore, the “state of impaired prediction of movement outcomes” can be associated with impairments in the coordinated complex (asynergia) and continuous repetitive (adiadochokinesis) movements.

Furthermore, the “state of impaired prediction of movement outcomes” requires a control through a delayed feedback mechanism. In this case, in the finger-nose test, the index finger deviates from the target (hypermetria). Correction occurs only after passing the target, resulting in a series of delayed corrections that overshoot and return, leading to large oscillations at the end (kinetic tremor). 12 Motor dysmetria seems to be closely linked to impaired predictions.

## VESTIBULOCEREBELLAR SYNDROME (VCS)

Within the cerebellum, three primary regions govern ocular movements and positioning: the flocculus and paraflocculus, the nodulus and uvula, and the dorsal vermis (lobules XI–XII), as shown in Figure 1 . The syndrome primarily manifests as abnormalities in ocular movements, including saccades and smooth pursuits, with stability arising from vestibulo-ocular reflex (VOR), and nystagmus. 24

Saccades are rapid eye movements that swiftly reposition the retinal image of an object onto the central fovea, which is crucial for maintaining high visual acuity in dynamic environments. The dorsal vermis and the fastigial nucleus exert primary control over saccades. Purkinje cells within the dorsal vermis facilitate the ipsilateral saccades while simultaneously inhibiting the contralateral ones. Consequently, lesions within the dorsal vermis result in hypometric ipsilateral and hypermetric contralateral saccades. 24 This clinical presentation can be attributed to the inhibitory influence of Purkinje cells upon neurons within the fastigial oculomotor region, which in turn project their axons contralaterally to the fastigial oculomotor region and, subsequently, to brainstem regions, including excitatory burst neurons. 25

Smooth pursuit movements are defined as slow, sustained eye movements that track moving objects within the visual field. Lesions within the flocculus/paraflocculus, which projects to the vestibular nuclei, nucleus prepositus hypoglossi, and interstitial nucleus of Cajal, impair this smooth pursuit tracking, particularly during sustained visual tracking. 24

The VOR is responsible for stabilizing gaze during head rotations. Lesions within the nodulus/uvula and flocculus/paraflocculus disrupt its coordination. The nodulus/uvula enhances the inherently low-frequency performance of the VOR, functioning as an integrator. 24 Conversely, the flocculus/paraflocculus regulates its amplitude and direction. 24 Given the inherent mechanical limitations of the labyrinth in transducing sustained motion, the integration of its signals with the cerebellum's within the vestibular nuclei significantly improves the accuracy of self-motion estimation. 24

Nystagmus can arise following lesions within the nodulus/uvula (downbeat, periodic alternating) or the flocculus/paraflocculus (gaze-evoked, downbeat, rebound). 11

A unified framework for understanding the aforementioned abnormal syndromes, particularly impaired saccades, pursuit, and VOR, can also be found within the cerebellar internal model theory. It hypothesizes that the cerebellum possesses an inherent internal model that can predictively calculate the current and desired positions of the eyeballs. 26

## CEREBELLAR COGNITIVE AFFECTIVE SYNDROME/SCHMAHMANN SYNDROME (CCAS/SS)

This syndrome is observed in cases with lesions in the cerebellar posterior lobe and vermis ( Figure 1 ). Patients exhibit deficits in executive functions, spatial cognition, linguistic processing, and emotional regulation. 8

Executive dysfunction encompasses impairments in planning, mental flexibility, abstract reasoning, working memory, and verbal fluency. Patients may also present with clinical features such as telegraphic speech, perseverative ideation, and mutism. 27 Impaired spatial cognition manifests as visuospatial disintegration, difficulties in drawing or copying diagrams, disorganized conceptualization of figures, impaired visuospatial memory, and simultanagnosia. 27 Linguistic difficulties include anomia, agrammatic speech, abnormal syntactic structures, and abnormal prosody, characterized by high-pitched, hypophonic whining. 27

Emotional dysregulation, particularly when lesions involve the vermis and fastigial nucleus, is a prominent feature of CCAS. The affective component of CCAS can be categorized into five neuropsychiatric domains: attentional control, emotional control, social skill set, autism spectrum disorders, and psychosis spectrum disorders. 27 Notably, within each domain, there exists a duality of symptoms: a positive, exaggerated symptom (hypermetria) and a negative, diminished symptom (hypometria). For instance, within the attentional control domain of CCAS/SS, individuals may exhibit either inattentiveness and hyperactivity, or ruminative thoughts and struggle with focusing. Similarly, within the emotional control domain, impulsivity or anergia can be observed. In the autism spectrum, individuals may display either stereotypical behaviors or exhibit avoidant behaviors. The psychosis spectrum may manifest illogical thought processes or lack of empathy, while the social skill set may be marked by anger or passivity. This duality contributes to Schmahmann's proposed concept of “dysmetria of thought.”

One of the most severe forms of CCAS/SS is cerebellar mutism, also known as posterior fossa syndrome, which typically occurs in children following surgery for midline cerebellar or intraventricular posterior skull base tumors. 28 Cerebellar mutism presents with a complex constellation of neurological and neurocognitive features, with a severe, albeit usually reversible, language disorder as the core feature. Language and speech impairments include apraxia, slowness of speech, reduced verbal fluency, and diminished spontaneous speech. 28

Recent research has highlighted the significance of autistic syndromes in understanding cerebellar cognitive and emotional control. It has been proposed that the core pathology in CCAS/SS is impaired predictive function in both the cognitive and emotional domains. Autism is characterized by impairments in cerebellar adaptive prediction, the process of generating expectations or predictions to rapidly adapt to changing stimuli or situations. 29 Adaptive prediction involves utilizing past experiences to infer intentions from the actions of others, predict what others might say, and infer the mental states of others, enabling individuals to swiftly modify their own behavior in response to the intentions of those around them. 30

Leggio and Molinari 31 proposed the “sequencing prediction” hypothesis. The hypothesis proposed transmission of an efferent copy of cognitive processes from the cerebral cortex to the cerebellum via a closed-loop pathway. The cerebellum then identifies the temporal sequence of these events and encodes them within the internal model. Consequently, the cerebellum compares novel inputs from the cerebral cortex with expected behavioral and sensory consequences. Furthermore, the cerebellum automatically fine-tunes cerebral cortical activity upon the detection of discrepancies. This theory aligns well with the concept of the internal model in motor control.

## CEREBELLAR RESERVE

A notable characteristic of the cerebellum is its capacity for functional recovery following damage. 32 This phenomenon is termed cerebellar reserve and can be attributed to several factors: the inherent redundancy of this organ's functional units, the convergent input via the extensive mossy fiber-parallel fiber network, and the diverse forms of synaptic plasticity. Consequently, it is hypothesized that functional units can compensate for damaged ones, and through synaptic plasticity, establish appropriate output pathways from previously underutilized cerebral inputs. Cerebellar reserve applies to CMS, VCS, and CCAS/SS. Patients may show a clinical compensation for the three syndromes.

## CLINICAL SCALES IN ATAXIOLOGY

**Figure 4:**
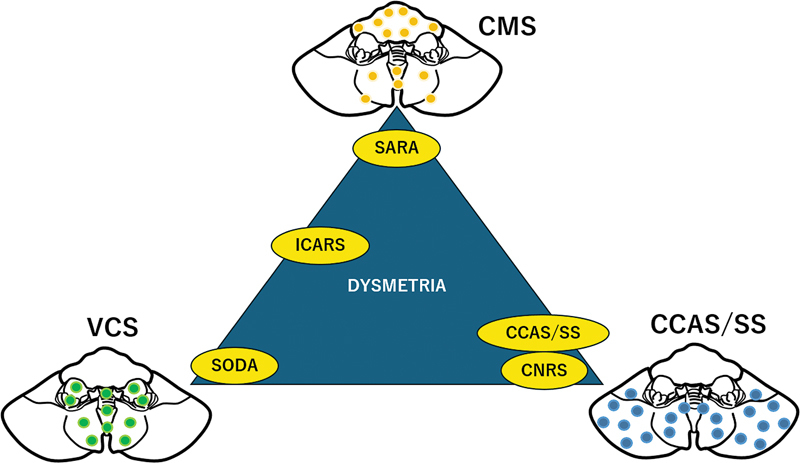
Abbreviations: CMS, cerebellar motor syndrome; VCS, vestibulocerebellar syndrome. The three cornerstones of cerebellar ataxiology and the clinical rating scales applied in daily practice. The three clinical syndromes are related to a symptom-lesion mapping: the sensorimotor cerebellum corresponds to lobules I–VI and VIII (orange circles), the cognitive cerebellum is located in lobules VI–IX (blue circles), and the vestibular cerebellum matches to lobules V–VII and IX–X (green circles). The clinical scale SARA assesses CMS, the SODA scale assesses VCS, and the Schmahmann rating scale assesses CCAS/SS. The ICARS scale assesses both CMS and VCS. The CNRS scale assesses the neuropsychiatric aspects of CCAS/SS.

Clinical scales remain the most robust instruments in clinical practice. 10 The most common ones for ataxia are the scale for the assessment and rating of ataxia (SARA) and international cooperative ataxia rating scale (ICARS) for CMS, scale for ocular motor disorders in ataxia (SODA) for VCS, and for CCAS/SS the Schmahmann rating and the recently described cerebellar neuropsychiatric rating (CNRS) scales ( Figure 4 ). 9 All of them fit with the classification into 3 clinical cerebellar syndromes and the location of cerebellar lesions. In all these scales, dysmetria is the core-feature.

In conclusion, the history of cerebellar neurology has been marked by attempts to reconcile its diverse neurological syndromes into a unified framework. The early work of Babinski, Holmes, and Garcin provided meticulous description of various motor symptoms. 1 3 4 6 20 Subsequent research extended this work by analyzing eye movement abnormalities in various cerebellar disorders. Moreover, in the 1990s, CCAS/SS emerged as a concept, encompassing a broad spectrum of cognitive and emotional disturbances, further enriching the field but also complicating the understanding of the neural system.

Studies on neuropsychiatry of the cerebellum also emerged, 9 bringing insights that offered a potential solution to this complexity. The homogeneous neural network was discovered within the cerebellar cortex in the 1960s, coupled with the elucidation of closed-loop connections between the cerebellum and other key brain regions (cerebrum, basal ganglia, brainstem) in the following years, it laid the groundwork for the principle of cerebellar unity and the concept of “universal cerebellar transform” proposed by Schmahmann. Dysmetria applies to motor, cognitive, and affective deficits.

Assuming the critical role of the cerebellum as an internal forward model that predicts the outcomes of motor, cognitive, and emotional processes, we propose that cerebellar signs and symptoms can be explained under a unified framework as disruptions in these predictive functions across all domains. The next crucial step includes the development of a comprehensive computational unified model that can accurately predict these outcomes.
